# De novo genome assembly of Ansell's mole-rat (*Fukomys anselli*)

**DOI:** 10.1093/g3journal/jkaf271

**Published:** 2025-11-11

**Authors:** Milica Bekavac, Raphael Coimbra, Veronica F Busa, Mikaela Behm, Rebecca E Wagner, Angela Goncalves, Sabine Begall, Michaela Frye, Duncan T Odom

**Affiliations:** German Cancer Research Center (DKFZ), Division of Regulatory Genomics and Cancer Evolution, Im Neuenheimer Feld 580, Heidelberg 69120, Baden-Württemberg, Germany; German Cancer Research Center (DKFZ), Division of Mechanisms Regulating Gene Expression, Im Neuenheimer Feld 280, Heidelberg 69120, Baden-Württemberg, Germany; Heidelberg University, Faculty of Biosciences, Grabengasse 1, Heidelberg 69117, Baden-Württemberg, Germany; German Cancer Research Center (DKFZ), Division of Regulatory Genomics and Cancer Evolution, Im Neuenheimer Feld 580, Heidelberg 69120, Baden-Württemberg, Germany; German Cancer Research Center (DKFZ), Division of Regulatory Genomics and Cancer Evolution, Im Neuenheimer Feld 580, Heidelberg 69120, Baden-Württemberg, Germany; German Cancer Research Center (DKFZ), Division of Mechanisms Regulating Gene Expression, Im Neuenheimer Feld 280, Heidelberg 69120, Baden-Württemberg, Germany; German Cancer Research Center (DKFZ), Division of Computational and Molecular Prevention, Im Neuenheimer Feld 580, Heidelberg 69120, Baden-Württemberg, Germany; German Cancer Research Center (DKFZ), Division of Mechanisms Regulating Gene Expression, Im Neuenheimer Feld 280, Heidelberg 69120, Baden-Württemberg, Germany; German Cancer Research Center (DKFZ), Division of Mechanisms Regulating Gene Expression, Im Neuenheimer Feld 280, Heidelberg 69120, Baden-Württemberg, Germany; Heidelberg University, Faculty of Biosciences, Grabengasse 1, Heidelberg 69117, Baden-Württemberg, Germany; German Cancer Research Center (DKFZ), Division of Computational and Molecular Prevention, Im Neuenheimer Feld 580, Heidelberg 69120, Baden-Württemberg, Germany; DKFZ Hector Cancer Institute at the University Medical Center Mannheim, Germany; Department of General Zoology, Faculty of Biology, University of Duisburg-Essen, Universitätsstr. 5, Essen 45117, North Rhine-Westphalia, Germany; German Cancer Research Center (DKFZ), Division of Mechanisms Regulating Gene Expression, Im Neuenheimer Feld 280, Heidelberg 69120, Baden-Württemberg, Germany; German Cancer Research Center (DKFZ), Division of Regulatory Genomics and Cancer Evolution, Im Neuenheimer Feld 580, Heidelberg 69120, Baden-Württemberg, Germany

**Keywords:** *Fukomys anselli*, genome assembly, nanopore, mole-rat, Ansell's mole-rat, Bathyergidae, Animalia

## Abstract

Ansell's mole-rat (*Fukomys anselli*) is an African rodent known for its subterranean lifestyle and unique phenotypic traits, including extreme longevity, magnetoreception, and a cooperative breeding social structure. Efforts to dissect the genetic architecture of these traits and to decipher their phylogenetic relationships within the broader African mole-rat family would greatly benefit from a reference-grade genome. Here, we report a first genome assembly of a male Ansell's mole-rat. By combining Oxford Nanopore Technologies long reads and Illumina short reads with Hi-C data, we generated a chromosome level assembly with a total length of 2.27 Gb, 412 scaffolds, and a scaffold N50 of 72.4 Mb. We identified 99.54% of expected genes and annotated 29,094 transcripts using RNA sequencing data. This high-quality de novo genome of *F. anselli* lays the foundation for dissecting the genetic and evolutionary basis of its extraordinary traits and resolving African mole-rat phylogeny.

## Introduction

Ansell's mole-rat (*Fukomys anselli*) is a subterranean rodent endemic to Zambia (Burda et al. 1999). Like other species within the African mole-rats family (Bathyergidae), Ansell's mole-rat lives in complex, narrow, self-excavated tunnels and has developed multiple morphological and physiological adaptations to this underground lifestyle (reviewed in Begall et al. 2021). The animals tolerate low levels of O_2_/high levels of CO_2_ and respond to hypoxia/hypercapnia with downregulation of free thyroid hormone T3 and erythropoiesis (Henning et al. 2024). Ansell's mole-rat demonstrates restricted visual capabilities (Němec et al. 2004; Wegner et al. 2006a) and hearing range (Gerhardt et al. 2017), but has a well-developed olfactory system (Caspar et al. 2022) and magnetoreception (Burda et al. 1990; Marhold et al. 1997; Wegner et al. 2006b; Caspar et al. 2020) for navigation through tunnels.

Ansell's mole-rats are exceptionally long-lived among rodents, with lifespans reaching up to 22 years (Dammann et al. 2022). They live in multigenerational family groups with a monogamous reproductive pair where most nonbreeders remain in the family and dispersal is delayed (Patzenhauerová et al. 2013; Garcia-Montero et al. 2016). Interestingly, lifespan within the *Fukomys* genus is notably shorter for nonbreeders compared to breeders, providing an opportunity to study gene regulation and expression that affects aging between closely related individuals (Dammann et al. 2011 , 2022; Sahm et al. 2021). However, current research efforts on the molecular mechanisms underlying longevity and social structures have been limited by a lack of high-resolution genomes for comparative studies within the *Fukomys* genus (Sahm et al. 2021). High-resolution genomes will unlock sequencing for variant calling, epigenomic analyses (commonly used in aging studies), integrative multiomics, and many other functional genomics approaches.

Extreme longevity is not unique to *F. anselli* within the Bathyergidae family. Notable examples of exceptionally long lifespan include the naked mole-rat (*Heterocephalus glaber*, maximum age 40 years) and Damaraland mole-rat (*Fukomys damarensis*, maximum age > 20 years) (Fang et al. 2014; Dammann et al. 2019; Buffenstein and Amoroso 2024; Ruby et al. 2024). These species have become important resources for research in cancer (Liang et al. 2010; Tian et al. 2013), hypoxia tolerance (Park et al. 2017), pain insensitivity (Park et al. 2008), and reproduction (Schmidt et al. 2014; Brieño-Enríquez et al. 2023). Yet, the genomes of *H. glaber* and *F. damarensis* remain the only available assemblies for the Bathyergidae family (Fang et al. 2014; Keane et al. 2014; Sokolowski et al. 2024).

Comparative genomics efforts resting on de novo transcriptomes have been used to analyze Bathyergidae divergence (Davies et al. 2015; Sahm et al. 2018b, 2021). However, these approaches are limited by: input data quality (Smith-Unna et al. 2016), a reliance on only the longest isoform for expression quantification (Davies et al. 2015; Sahm et al. 2018a, 2021), unreliable expression estimates due to fragmented contiguous sequences (Hsieh et al. 2019) or short transcript lengths (Wu et al. 2018), incomplete transcript assembly (Ungaro et al. 2017), and species-specific performance of assemblers (Hölzer and Marz 2019). Harnessing high-quality whole-genome assemblies from multiple Bathyergidae species empowers comprehensive interspecies analyses and eliminates the barriers imposed by transcriptome-only comparisons.

Ansell's mole-rat was first formally described in Burda et al. (1999) as *Cryptomys anselli*, with a distinguishing karyotype of 2*n* = 68. Further phylogenetic and karyotypic evidence led to the taxonomic separation of *Fukomys* from the *Cryptomys* genus (Kock et al. 2006). Nevertheless, the Bathyergidae phylogeny remains debated (Visser et al. 2019) (see below). Prior efforts have leveraged 12S rRNA, TTR intron I, and mitochondrial cytb sequencing to genetically resolve phylogenetic relationships (Faulkes et al. 2004; Ingram et al. 2004; Van Daele et al. 2007). Resolving the phylogenetic relationships within Bathyergidae will require broader taxon sampling, particularly from additional genera, but adding a third high-quality genome represents significant progress. The resolution of such analyses depends on the contiguity and annotation quality of all included genomes, but cross-species analysis power and precision will continue to improve as additional bathyergid assemblies become available.

Here, we report the first chromosome-scale genome of a male *F. anselli*, assembled using Oxford Nanopore Technologies (ONT) long-read, Illumina short-read, and Hi-C sequencing. This high-quality genome provides the foundation for uncovering the genetic basis of Ansell's mole-rat's remarkable traits, including longevity, hypoxia tolerance, and magnetoreception.

## Materials and methods

### Sample collection

Animals were housed at the University of Duisburg-Essen (approved by permit no. 32-2-1180-71/328 Veterinary Office of the City of Essen) in a humidity and temperature-controlled room with a 12L:12D cycle. Ambient temperature was kept constant at 24 ± 1 °C with relative humidity of 40% to 50%. The animals were fed raw carrots and potatoes three times per week as staple food, supplemented with cereals and apples once per week and lettuce on an irregular basis. No free water was needed. They were kept in family groups in glass terraria ranging in size (W × L × H) from 45 × 70 × 40 to 60 × 140 × 40 cm, depending on family size. The terraria were filled with sawdust, and plastic or wooden tubes enriched the terraria. Flower pots served as nests, supplemented with hay and paper strips as nesting material.

The animals were put under deep anesthesia with an intramuscular injection of 12 mg/kg ketamine (Ceva GmbH) and 5 mg/kg xylazine (Ceva GmbH) and then decapitated. We collected organs for long-read sequencing, short-read sequencing, Hi-C sequencing, and RNA sequencing from a 21-mo-old nonbreeder male. Liver, spleen, testes, calf muscle, kidney, heart, and lung were dissected and flash frozen by placing them directly in liquid nitrogen. Flash frozen brain and skin from two other nonbreeder males, 12- and 19-mo-old individuals, respectively, were also used for RNA sequencing experiments.

### Nanopore long-read sequencing

Frozen liver was pulverized using CP02 CryoPrep Automated Dry Pulverizer (Covaris) and genomic DNA was isolated using the Puregene Tissue kit (Qiagen) according to manufacturer's instructions, with the following modifications when using the ONT Ultra-Long DNA Sequencing Kit V14 for subsequent library preparation: the final elution buffer was changed to EEB buffer from ONT and the first incubation for dissolving DNA decreased to 56 °C for 20 min. One DNA extraction was performed using the Nanobind PanDNA kit (PacBio), according to the manufacturer's instructions, with a change in the initial tissue processing (CryoPrep to disrupt the tissue) and the final elution buffer to EEB buffer (ONT). The DNA was quantified using the Qubit dsDNA BR Assay kit (Invitrogen) and the DNA integrity was verified with Genomic DNA ScreenTape Assay on a TapeStation (Agilent Technologies). Libraries were prepared using the ONT Ultra-Long DNA Sequencing Kit V14 (SQK-ULK114) (starting from the Tagmentation reaction) or ONT Ligation Sequencing kit V14 (SQK-LSK114). Sequencing was performed on one MinION flow cell on a GridION instrument and 7 PromethION flow cells (R10.4.1), which generated a total of 157.41 GBases.

### Illumina short-read sequencing

DNA was isolated from a frozen spleen fragment using AllPrep DNA/RNA/Protein Mini kit (Qiagen) according to the manufacturer's instructions. The DNA was quantified using the Qubit dsDNA BR Assay kit (Invitrogen) and its integrity was verified with Genomic DNA ScreenTape Assay on a TapeStation (Agilent Technologies). The library was prepared using the Illumina DNA PCR-Free Library Prep kit, according to the manufacturer's instructions. It was quantified with Qubit ssDNA Assay Kit (Invitrogen) and sequenced on Illumina NovaSeq X Plus instrument in a 2 × 150 bp configuration, which generated 400.81 GBases of data.

### Hi-C sequencing

An Arima High Coverage HiC kit (Arima Genomics) was used to generate proximally ligated DNA according to the manufacturer's protocol (A160162 v01), with the following modifications: the liver was pulverized with CP02 CryoPrep Dry Pulverizer (Covaris) instead of mortar and pestle; step 2 of the proximal ligation was extended to 20 min. A library compatible with Illumina sequencing was prepared with the Arima Library Prep Module (Arima Genomics), according to the protocol for Arima High Coverage HiC kit (Document number: A160186 v02). The concentration and size distribution of the library were measured with Qubit 1X dsDNA High Sensitivity Assay kit (Invitrogen) and D5000 ScreenTape Assay on TapeStation (Agilent Technologies), respectively. The library was sequenced on two lanes of an Illumina NovaSeq 6000 instrument in 2 × 250 bp mode. In total, 355.93 GBases of data were generated.

### RNA sequencing

Liver, spleen, testes, calf muscle, kidney, heart, lung, and brain were pulverized using CP02 CryoPrep Dry Pulverizer (Covaris). The tissue powder was placed in tubes with metal beads and TRIzol (Invitrogen) and homogenized with TissueLyser (Qiagen). The debris was removed by centrifugation and total RNA was extracted with Direct-zol RNA MiniPrep kit (Zymo Research) according to manufacturer's instructions. The RNA quality was assessed with High Sensitivity RNA ScreenTape Assay on a TapeStation (Agilent Technologies) and quantity was measured with Qubit RNA BR Assay kit (Invitrogen). Libraries were prepared using the Illumina Stranded Total RNA Prep, Ligation with Ribo-Zero Plus kit according to the manufacturer's instructions. The quality of the libraries was assessed on a D5000 ScreenTape Assay on a TapeStation (Agilent Technologies) and their concentration was measured with Qubit 1X dsDNA High Sensitivity Assay kit (Invitrogen). Pooled libraries were sequenced on the Illumina NovaSeq X Plus instrument in 2 × 100 bp mode.

For skin RNA-seq, total RNA was extracted from abdomen skin tissue of a single *F. anselli* individual. The tissue was homogenized using a mechanical tissue homogenizer (POLYTRON), and RNA was isolated using a combination of TRIzol (Invitrogen) and the mirVana miRNA Isolation Kit (Thermo Fisher Scientific). RNA quantity and quality were assessed using the Qubit RNA BR Assay Kit (Invitrogen) and the Bioanalyzer RNA 6000 Nano Assay (Agilent Technologies). To eliminate genomic DNA contamination, the RNA was treated with TURBO DNase (Invitrogen), followed by purification using the RNA Clean & Concentrator kit (Zymo Research). RNA-seq libraries were prepared using the TruSeq Stranded Total RNA Library Prep Kit (Illumina) with IDT for Illumina TruSeq DNA/RNA Unique Dual Indexes (UDI). Sequencing was carried out on one Illumina NextSeq 550 lane using a 150 bp paired-end configuration.

### Assembly

Prior to assembly, we estimated the genome size and repeat coverage based on *k*-mer frequencies in the short-read sequencing via Jellyfish v2.3.1 and GenomeScope v2.0.1 (Marçais and Kingsford 2011; Ranallo-Benavidez et al. 2020). We base-called the long-read sequencing using the sup model of Dorado v.0.9.0 (ONT PLC. Public License, v. 1.0), then assembled and performed two rounds of polishing on the long-read sequencing via Flye v2.9.5-b1801 (Kolmogorov et al. 2019) using a GenomeScope-based genome size estimate of 2.3 Gb and the argument --nano-hq. Although we attempted further polishing using medaka v2.0.1, this was detrimental to genome assembly based on multiple quality assessment metrics such as error rate, *k*-mer completeness, contig N50, and gene completeness, so this additional polishing was omitted in the final version. We leveraged the short-read sequencing to correct base errors (SNVs/indels) in the polished assembly via NextPolish v1.4.1 (Hu et al. 2020).

We then used purge_dups v1.2.6 (Guan et al. 2020) to remove haplotigs and overlaps. Only haplotigs and overlaps at the end of contigs were selected for removal to avoid accidental deletion of true duplications in the middle of contigs. We used the BLAST+ v2.16.0 executable blastn (Camacho et al. 2009) to identify possible contamination, minimap2 v2.28 (Li 2021) and samtools v1.21 (Danecek et al. 2021) to generate duplicate-marked short-read alignments to the purged assembly, and BlobToolkit v4.4.0 (Challis et al. 2020) to calculate and visualize contig coverage and GC content. Contigs were removed as contamination if they returned no hits (6,715 contigs) or were assigned to orders other than Rodentia and did not match any known mole-rat sequences (8 contigs). Additionally, all contigs shorter than 1 kbp (182 contigs) were removed prior to scaffolding.

Hi-C sequencing was cleaned via fastp v0.24.0 (Chen et al. 2018) to trim adapters and polyG sequencing artifacts and to discard low-quality reads and reads shorter than 36 bp, then processed and aligned to the cleaned contigs via a modified Arima Genomics mapping pipeline (doc A160156 v03 January 2024). Hi-C scaffolding was performed using YaHS v1.2.2 (Zhou et al. 2023), and the assembly was manually curated for misjoins, missed joins, translocations, and inversions using Juicebox with Assembly Tools v2.17.0 (Durand et al. 2016; Dudchenko et al. 2018). The final, curated assembly was visualized for synteny to *F. damarensis* (DMR_v1.0_HiC) via JupiterPlot v1.1 (Chu 2018).

### Quality assessment

We quantified the quality of our multiple long-read sequencing runs using NanoComp v1.24.2 and NanoPlot v1.43.0 (De Coster and Rademakers 2023) (Supplementary Fig. 1a, Supplementary Table 1).

We employed QUAST v5.3.0 (Gurevich et al. 2013) to measure assembly contiguity; Mercury with Meryl v1.4.1 (Rhie et al. 2020) to measure consensus quality (QV), error rate, and *k*-mer completeness; and compleasm v0.2.6 (Huang and Li 2023) to measure assembly completeness based on the “glires_odb10” gene set at all steps of the assembly pipeline to track assembly progress. We also used these tools to compare our de novo Ansell's mole-rat assembly to other available Bathyergidae genomes (Tables 1 and 2).

**Table 1.** Contiguity of available African mole-rat genomes.

	*F. anselli* (This assembly)	*F. damarensis* (DMR_v1.0_HiC)	*H. glaber* (HetGla_female_1.0)	*H. glaber* (mHetGlaV3)
# scaffolds	412	73,969	4,229	75
# scaffolds ≥ 10 Mbp	34	39	88	30
Total length	2,274,106,617	2,334,375,022	2,618,204,639	2,474,750,198
N50	72,399,386	62,586,000	20,532,749	97,315,161
N90	40,322,336	37,171,693	5,125,547	53,093,233
auN	80,188,288	66,455,256.8	22,553,264.2	94,268,059.9
L50	12	15	42	11
L90	28	34	142	25
GC (%)	40.43	40.34	40.21	39.96
*N*'s/100 kbp	1.45	2,072.24	11,589.37	71.12

**Table 2.** Completeness estimates of available African mole-rat genomes.

	*F. anselli* (This assembly)	*F. damarensis* (DMR_v1.0_HiC)	*H. glaber* (HetGla_female_1.0)	*H. glaber* (mHetGlaV3)
Single-copy complete	99.09%, 13,673	95.88%, 13,230	97.59%, 13,466	96.42%, 13,304
Duplicated complete	0.45%, 62	0.39%, 54	1.40%, 193	1.33%, 183
Fragmented	0.10%, 14	1.96%, 270	0.34%, 47	0.33%, 45
Missing	0.36%, 49	1.77%, 244	0.67%, 92	1.93%, 266

Short-read RNA-seq of all nine tissues was processed via fastp v0.24.0 (Chen et al. 2018) to trim adapters and polyG sequencing artifacts and to discard low-quality reads and reads shorter than 36 bp (Supplementary Fig. 1b). FastQ Screen v0.16.0 (Wingett and Andrews 2018) was used to validate that the library predominantly aligns with the available *F. damarensis* genome rather than potential contaminant genomes (Supplementary Fig. 1c). MultiQC v1.27.1 (Ewels et al. 2016) was used to visualize the RNA-seq quality.

OMArk v0.3.1 (Nevers et al. 2025) provided a proteome completeness and consistency assessment.

### Annotation

Repeats and transposable elements were identified and annotated using Earl Grey v5.1.1 (Baril et al. 2024) based on a de novo repeat library and known *F. damarensis* repeats from the Dfam database v3.7 (Hubley et al. 2016). Identified repeats were soft masked prior to gene annotation using Earl Grey.

Short-read RNA-seq derived from nine different tissues was aligned to the assembled scaffolds using STAR v2.7.11b (Dobin et al. 2013) in two-pass mode for splice-aware mapping. We then applied BRAKER v3.0.8 (Hoff et al. 2019; Gabriel et al. 2024) for structural gene annotation using the aligned RNA-seq along with protein sequences derived from OrthoDB v12 vertebrates (Tegenfeldt et al. 2025) and from 41 NCBI RefSeq annotated rodent species (Supplementary Table 2). Gene identification was optimized by assigning the argument busco_lineage to glires_odb10. Given the weak cross-species conservation of many noncoding RNAs and the absence of a reliable validation set, we restricted annotation to protein-coding genes.

### De novo assembly and annotation of the mitochondrial genome

The mitogenome was assembled using the short-read sequencing via GetOrganelle v1.7.7.1 (Jin et al. 2020) using the *F. damarensis* (NC_027742.1) and *H. glaber* (NC_015112.1) mitogenomes as seeds. Next, we applied MitoAnnotator v4.0.9 (Zhu et al. 2023) to annotate the newly assembled mitogenome. The annotated mitochondrial features were visually inspected and start/end positions were corrected when necessary based on comparisons with *F. damarensis* and *H. glaber* mitochondrial gene sequences and annotations. Modified OrganellarGenomeDRAW v1.3.1 (Greiner et al. 2019) outputs were used for visualization.

To confirm the mitogenome assembly using long-read ONT sequencing, potential mitochondrial reads were first extracted by mapping to *F. damarensis* mitogenome via minimap2 v2.28 (Li 2021). Reads shorter than 1 kb or longer than 20 kb were excluded. The remaining reads were compared to the *F. damarensis* reference mitogenome using BLAST+ v2.16.0 (Camacho et al. 2009) and were retained only if they covered at least 70% of the reference, normalized by the subject (read) length. We assembled and polished the mitogenome from filtered reads via Flye v2.9.5-b1801 (Kolmogorov et al. 2019); this resulted in a single, circular contig. Error correction was performed using short reads trimmed with fastp v0.24.0 (Chen et al. 2018) via Pilon v1.24 (Walker et al. 2014).

To verify the identity of our mitogenome, the final assembly sequence was aligned to four publicly available 12S rRNA sequences from different *F. anselli* individuals (AY427022-AY427025) (Ingram et al. 2004) using the L-INS-i algorithm in MAFFT v7.526 (Katoh and Standley 2013).

## Results and discussion

We de novo assembled and annotated the genome of a male Ansell's mole-rat (*F. anselli*) using multiple modalities. Briefly, we performed Oxford Nanopore long-read sequencing (ONT) using DNA isolated from liver to obtain a whole-genome coverage of 46× and Illumina short-read sequencing (WGS) using DNA isolated from spleen to obtain a whole-genome coverage of >175×. We used Flye to assemble the ONT and NextPolish to correct base errors in the sequence using WGS (Kolmogorov et al. 2019; Hu et al. 2020). We then cleaned contigs using purge_dups to remove haplotigs and BLASTn to identify and remove potential contamination (Supplementary Fig. 2a) (Camacho et al. 2009; Guan et al. 2020). This cumulatively produced a 2.41 Gb genome across 7,581 contigs with an N50 of 19.8 Mb (Supplementary Fig. 2b, Supplementary Table 3).

**Fig. 1.**
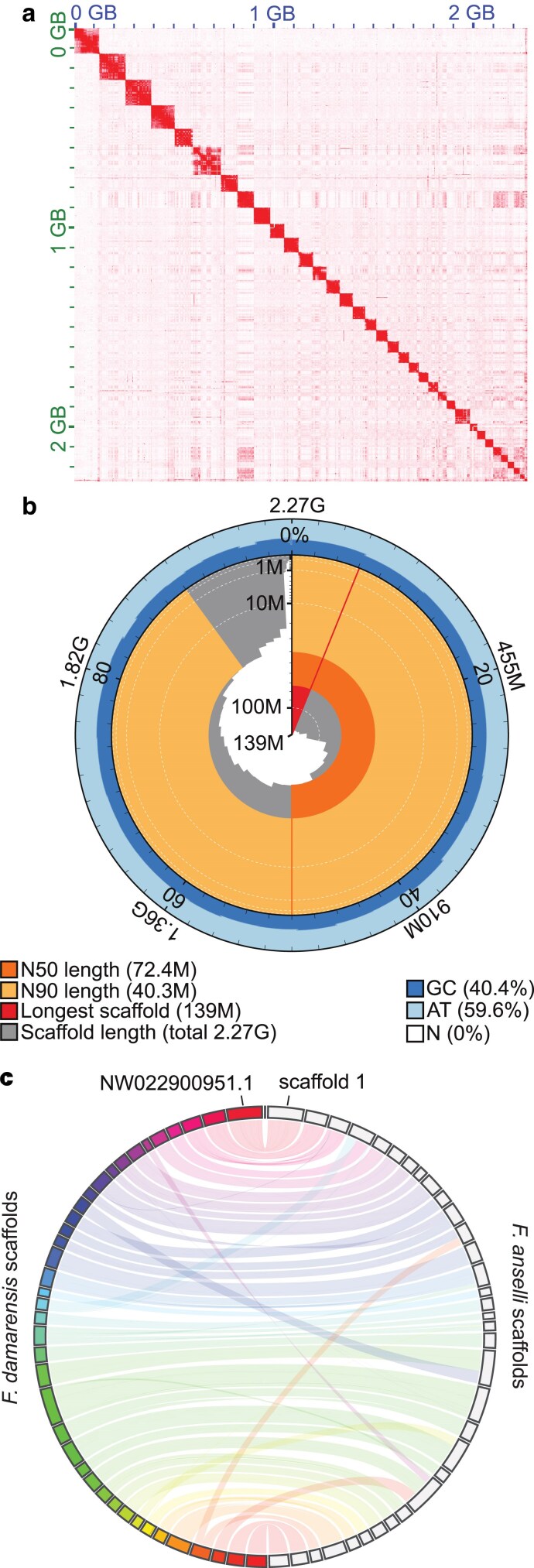
Chromosome-level *F. anselli* genome assembly. a) Hi-C read contact map following final scaffold curation. The shading intensity corresponds to the number of contacts. b) Snail plot depicting the length and GC content of scaffolds. c) Circos plot depicting synteny between *F. damarensis* (left, colorful) and *F. anselli* (right, grey) scaffolds for all *F. damarensis* scaffolds longer than 10 Mb and for *F. anselli* scaffolds constituting 99% of the genome. A fully annotated circos plot with scaffold numbers can be found in Supplementary Fig. 3.

Sequence alignment of Hi-C data yielded 13.3 million unique inter-contig reads (Supplementary Table 4), which were leveraged by YaHS to create a scaffolded genome that was manually curated using Juicebox and Assembly Tools (Fig. 1a, Supplementary Fig. 2c) (Durand et al. 2016; Zhou et al. 2023). The final de novo Ansell's mole-rat assembly is 2.27 Gb across 412 scaffolds with an N50 of 72.4 Mb and L50 of 12 (Fig. 1b, Table 1). This genome length is similar to expectations based on the 2.23 Gb estimated from *k*-mers by GenomeScope (Ranallo-Benavidez et al. 2020) and a previously assembled 2.3 Gb *F. damarensis* genome (DMR_v1.0_HiC) (Fang et al. 2014).

Our highly contiguous *F. anselli* genome is the first chromosome-level *Fukomys* genome and demonstrates ortholog completeness superior to other currently available resources. Gene analysis using compleasm (Huang and Li 2023) identified 99.54% ortholog completeness within the glires lineage, which represents all lagomorphs and rodents (Table 2). Prior research in the *Fukomys* genus has relied on incomplete and extremely fragmented genomes; for instance, *F. mechowii* and *F. micklemi* have only been analyzed using short-read RNA-seq-derived de novo transcriptomes, both of which contained approximately 20,000 contigs with assembly completeness of 93.7% and 93.4%, respectively, estimated by BUSCO (Sahm et al. 2018b). Although there is an available Hi-C-scaffolded *F. damarensis* genome, the best published resource is composed of 73,969 scaffolds with a completeness estimate of 96.27% (Tables 1 and 2).

As expected, *F. anselli* demonstrates a high degree of synteny with *F. damarensis* (Van Daele et al. 2007): the 34 largest *F. anselli* scaffolds (each larger than 10 Mb) represent 99% of the genome and correspond to all 39 *F. damarensis* scaffolds larger than 10 Mb (Fig. 1c, Supplementary Fig. 3). Although some *F. damarensis* scaffolds are split across *F. anselli's* scaffolds and vice-versa (e.g. the intersecting bands in Fig. 1c), there are few nonsyntenic regions. Synteny with the *F. damarensis* scaffold NW_022900951.1, which is annotated for known X-linked genes such as Ar, Dmd, Fmr1, and Mecp2, suggests that the largest scaffold in the *F. anselli* genome, scaffold 1, is the X chromosome (Fig. 1c, Supplementary Fig. 3). This is corroborated by the scaffold's depleted sequencing coverage, consistent with X chromosome hemizygosity in our male individual (Supplementary Fig. 2d). There have been reports that the chromosomal complement of *F. anselli*—in particular the sex chromosomes—may be more complex than could be captured by our single-individual reference XY genome (Burda et al. 1999). Although all karyotyped *F. anselli* individuals have been found to have a diploid chromosome number of 2*n* = 68, there appears to be variation in the size and centromeric positioning among both the sex and autosomal chromosomes. Indeed, the two X chromosomes in female karyotypes are often heteromorphic. Thus, to fully capture the standing structural variation among Ansell's mole-rat may require sequencing of additional individuals of both sexes.

### Mitochondrial genome

**Fig. 2.**
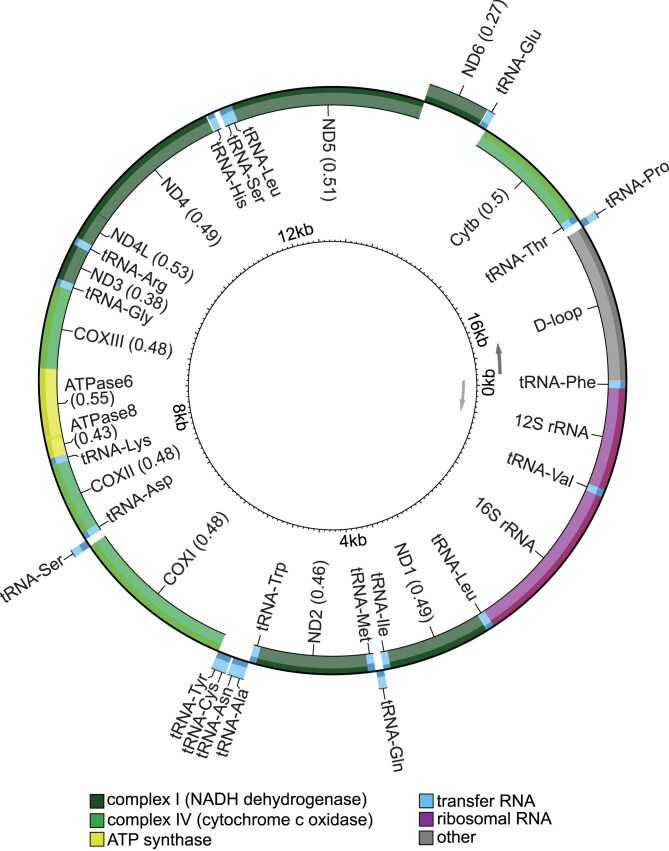
*F. anselli* mitogenome annotation. Genes are shown on the outside or inside of the circle according to transcriptional orientation. *F. anselli* has an expanded D-loop compared to other known mole-rat mitogenomes.

We assembled the mitochondrial genome using GetOrganelle based on *H. glaber* and *F. damarensis* mitochondrial assemblies and annotated it using MitoAnnotator (Jin et al. 2020; Zhu et al. 2023). Consistent with other annotated mole-rat genomes, we identified the control region, 13 genes, 2 rRNAs, and 22 tRNAs in the 17,006 bp *F. anselli* mitogenome (Fig. 2). The Ansell's mole-rat's mitogenome is larger than that of other closely related species: 16,372 bp in *F. damarensis* and 16,386 bp in *H. glaber.* This difference is mostly attributable to a longer control region (D-loop). To verify that this discrepancy is not due to artifacts of the assembly method, we independently assembled the mitogenome from ONT reads (see Materials and Methods), which generated a sequence differing by only one nucleotide (data not shown). Moreover, the alignment of the assembled mitogenome's 12S rRNA region with four publicly available *F. anselli* 12S rRNA sequences revealed high sequence identity (Ingram et al. 2004). The alignments contained no gaps and few nucleotide differences, which are consistent with expected intraspecific variation (data not shown).

### Repetitive regions and transposable elements

**Fig. 3.**
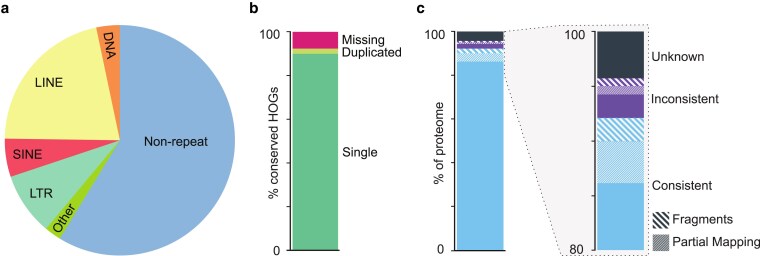
Repetitive regions and proteome. a) Proportion of the genome composed of repetitive regions and transposable elements. DNA, DNA transposon; LINE, long interspersed nuclear element; SINE, short interspersed nuclear element; LTR, long terminal repeat retrotransposon. b) Proteome completeness. HOG, hierarchical orthologous gene. c) Proteome consistency within the hystricomorph rodent HOG family.

Repeats and transposable elements were identified and annotated using Earl Grey (Baril et al. 2024). Repetitive elements span a total of 41.3% of the *F. anselli* genome, with retroelements (LINEs, SINEs, Penelope class, and LTR elements) constituting 35.6%, DNA transposons 3.3%, and simple repeats 1.0% (Fig. 3a, Table 3, Supplementary Table 5, Supplementary Fig. 4). The genomic fraction of repetitive regions is higher than that observed in *H. glaber* (28.9% in HetGla_female_1.0) or *F. damarensis* (30.3% in DMR_v1.0_HiC).

**Table 3.** *Fukomys anselli* repetitive regions and transposable elements.

	Coverage (bp)	Copy number	% Genome coverage	TE family count
DNA	74,913,617	408,765	3.2942	397
Rolling circle	140,571	673	0.0062	5
Penelope	9,165	48	0.0004	1
LINE	488,312,729	1,503,433	21.4727	265
SINE	121,651,153	1,137,637	5.3494	58
LTR	200,486,946	748,964	8.8161	593
Other (Simple repeat, microsatellite, RNA)	27,836,643	601,114	1.2241	12,474
Unclassified	24,898,076	173,619	1.0949	156
Total repeats	938,248,900	…	41.2579	…
Non-repeat	1,335,857,717	…	58.7421	…

Our assembly of *F. anselli* used both short-read and ONT long-read sequencing; in contrast, both the *H. glaber* and *F. damarensis* genomes (HetGla_female_1.0; DMR_v1.0_HiC) were assembled using only short-read whole-genome sequencing, which can fail to capture the full extent of the repetitive genome (Lewis et al. 2016; Platt et al. 2016). We therefore compared our genome to a recent naked mole-rat genome (mHetGlaV3) assembled using similar long-read sequencing data. Indeed, this naked mole-rat genome reports a repetitive element composition strikingly similar to our findings in *F. anselli*: cumulatively 40.4% of the genome, with 36.2%, 2.6%, and 1.0% retroelements, DNA transposons, and simple repeats, respectively (Sokolowski et al. 2024).

### Gene structure annotation

Protein-coding regions of the curated and repeat-softmasked *F. anselli* assembly were annotated using BRAKER (Hoff et al. 2019; Gabriel et al. 2024). We generated RNA-seq from nine different tissues (liver, spleen, testes, calf muscle, kidney, heart, lung, brain, back skin) and combined them with protein sequences annotated in closely and distantly related species (Supplementary Table 2). In total, we identified 29,094 transcripts across 18,256 predicted genes.

Assessment of proteome completeness via OMArk (Nevers et al. 2025) affirmed that *F. anselli* belongs to the hystricomorph rodent hierarchical orthologous gene (HOG) family. The *F. anselli* annotated proteome contains 92.35% of the 13,570 conserved Hystricomorpha HOGs and no contaminant sequences (Table 4, Fig. 3b and c). Further, among the 18,256 *F. anselli* predicted protein-coding genes, 92.18% are consistent with known gene families within Hystricomorpha; only 4.23% do not correspond to proteins in a known gene family (Table 4, Fig. 3c). The species calculated to be most consistent with the HOGs in the annotated genome is *F. damarensis*, with 95.77% concordance; this estimate provides validation of phylogenetic proximity of these two species.

**Table 4.** *Fukomys anselli* proteome completeness and consistency.

Completeness	
Conserved HOGs (Hystricomorpha)	**13,570**
Single	**12,210** (**89.98%)**
Duplicated	**322** (**2.37%)**
Unexpected	279 (2.06%)
Expected	43 (0.32%)
Missing	**1,038** (**7.65%)**

Consistency	
Total proteins	**18,256**
Consistent	**16,828** (**92.18%)**
Partial hits	697 (3.82%)
Fragmented	390 (2.14%)
Inconsistent	**656** (**3.59%)**
Partial hits	136 (0.74%)
Fragmented	131 (0.72%)
Contaminants	**0** (**0.00%)**
Unknown	**772** (**4.23%)**

Bold values sum to 100%.

### Ongoing discussion of phylogenetic placement

There has been a long-standing debate regarding the taxonomic placement of several *Fukomys* species, including *F. anselli* (Faulkes et al. 2004, 2017; Ingram et al. 2004; Van Daele et al. 2007; Šumbera et al. 2023). Phenotypic and genotypic considerations may lead to re-designation of *F. anselli* (personal communication from Radim Šumbera). Regardless of potentially altered nomenclature, this genome assembly is utilizable for investigating *Fukomys* species with the karyotype of 2*n* = 68 as well as closely related species and hybrid animals.

In summary, our de novo genome of the Ansell's mole-rat demonstrates chromosome-level contiguity and near-perfect gene completeness. We provide a novel annotated mitogenome, well-resolved repetitive regions and transposable elements, and a thorough protein-coding transcript annotation derived from nine diverse tissues. Given its completeness and quality, the *F. anselli* genome is comparable or superior to other available rodent genomes, providing a powerful resource to study the organismal phenotypes of Ansell's mole-rat and resolving Bathyergidae phylogeny.

## Supplementary Material

jkaf271_Supplementary_Data

## Data Availability

The genome assembly and raw sequencing reads (WGS of ONT long reads and Illumina short reads, Hi-C, and RNA-seq) generated in this study are deposited at NCBI under the BioProject PRJNA1240209. The nucleotide sequences of the Ansell's mole-rat nuclear and mitochondrial genomes assembled in this study are available at GenBank under the accessions JBPETH000000000 and PV670003, respectively. The gene structure and repeat annotation files associated with the Ansell's mole-rat genome assembly are available on Zenodo (https://doi.org/10.5281/zenodo.15350947). The code used to process, assemble, and annotate the Ansell's mole-rat genome is also available on Zenodo (https://doi.org/10.5281/zenodo.15489638). Nuclear and mitochondrial genome sequences of other mole-rat species used in this study are publicly available and include: Damaraland mole-rat genome assembly DMR_v1.0_HiC (NCBI RefSeq: GCF_012274545.1) (Fang et al. 2014) and mitochondrion (NCBI RefSeq: NC_027742.1); naked mole-rat genome assemblies HetGla_female_1.0 (NCBI RefSeq: GCF_000247695.1) (Keane et al. 2014) and mHetGlaV3 (ENA: GCA_964261345) (Sokolowski et al. 2024), and mitochondrion (NCBI RefSeq: NC_015112.1). Proteomes used in this study are publicly available, and their corresponding accession numbers are listed in Supplementary Table 2. Supplemental material available at G3 online.
