# Correction to: De novo genome assembly of Ansell's mole-rat (*Fukomys anselli)*

**DOI:** 10.1093/g3journal/jkag198

**Published:** 2026-07-23

This is a correction to: Milica Bekavac, Raphael Coimbra, Veronica F Busa, Mikaela Behm, Rebecca E Wagner, Angela Goncalves, Sabine Begall, Michaela Frye, Duncan T Odom, De novo genome assembly of Ansell's mole-rat (*Fukomys anselli*), *G3 Genes|Genomes|Genetics*, Volume 16, Issue 1, January 2026, jkaf271, https://doi.org/10.1093/g3journal/jkaf271

In the originally published version of this manuscript, an institutional affiliation was omitted for the sixth co-author.

“DKFZ Hector Cancer Institute at the University Medical Center Mannheim, Germany” has been added and assigned to Angela Goncalves.

The subsequent affiliation has been re-numbered accordingly.

