# Supplementary material for: De novo genome assembly of Ansell's mole-rat (Fukomys anselli)
Source: G3 (Bethesda). 2025 Nov 11;16(1):jkaf271. doi: 10.1093/g3journal/jkaf271 (PMC12774600; doi:10.1093/g3journal/jkaf271)
Supplement: jkaf271_Supplementary_Data [file jkaf271_supplementary_data.zip › Figure_S1_G3-2025-406291.pdf]

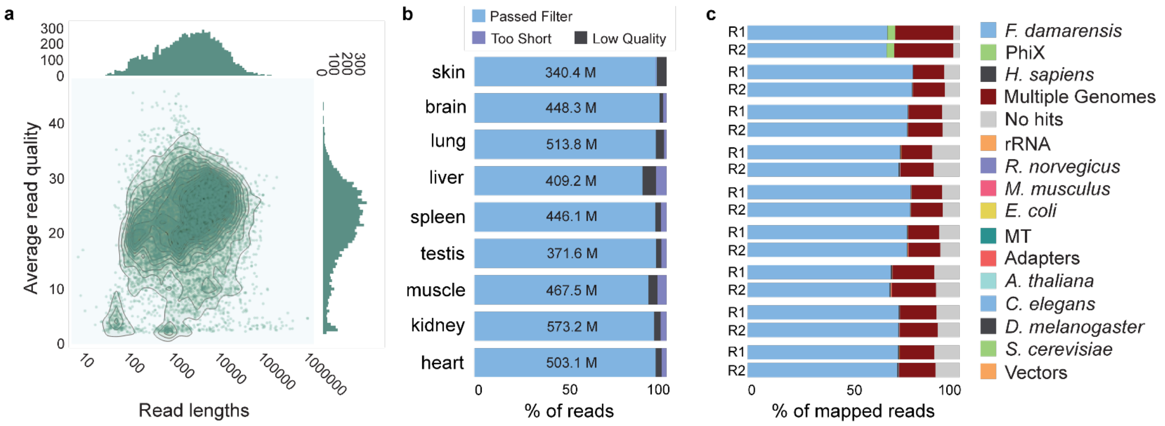

Figure S1: Sequencing quality control metrics. a) Pooled nanopore sequencing length and quality, per read. b) RNA-seq read quality. Numbers of reads that passed filter are indicated on the bars. c) Predicted genome alignment for nine tissues.
