# Supplementary material for: De novo genome assembly of Ansell's mole-rat (Fukomys anselli)
Source: G3 (Bethesda). 2025 Nov 11;16(1):jkaf271. doi: 10.1093/g3journal/jkaf271 (PMC12774600; doi:10.1093/g3journal/jkaf271)
Supplement: jkaf271_Supplementary_Data [file jkaf271_supplementary_data.zip › Figure_S2_G3-2025-406291.pdf]

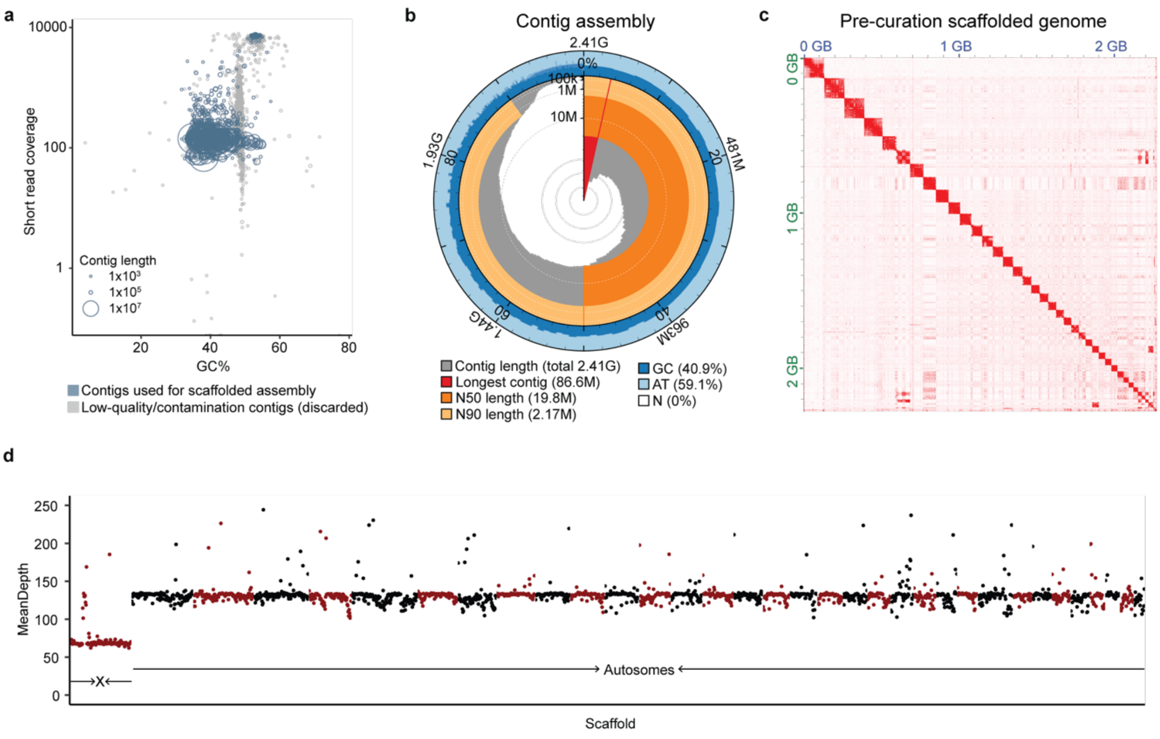

Figure S2: Intermediate steps in assembling the *Fukomys anselli* genome. a) Contig length, coverage, and GC content among assembled contigs. Grey contigs were identified as low-quality or contamination and so were not included in the final scaffolded assembly. b) Snail plot depicting the length and GC content of all contigs following purging of contamination contigs. c) Hi-C contact map of scaffolded genome prior to curation. The saturation of red corresponds to the number of contacts. d) Hi-C read coverage of the curated assembly along 1Mb bins. The average read depth is truncated at 250x coverage.
