# Supplementary material for: De novo genome assembly of Ansell's mole-rat (Fukomys anselli)
Source: G3 (Bethesda). 2025 Nov 11;16(1):jkaf271. doi: 10.1093/g3journal/jkaf271 (PMC12774600; doi:10.1093/g3journal/jkaf271)
Supplement: jkaf271_Supplementary_Data [file jkaf271_supplementary_data.zip › Figure_S3_G3-2025-406291.pdf]

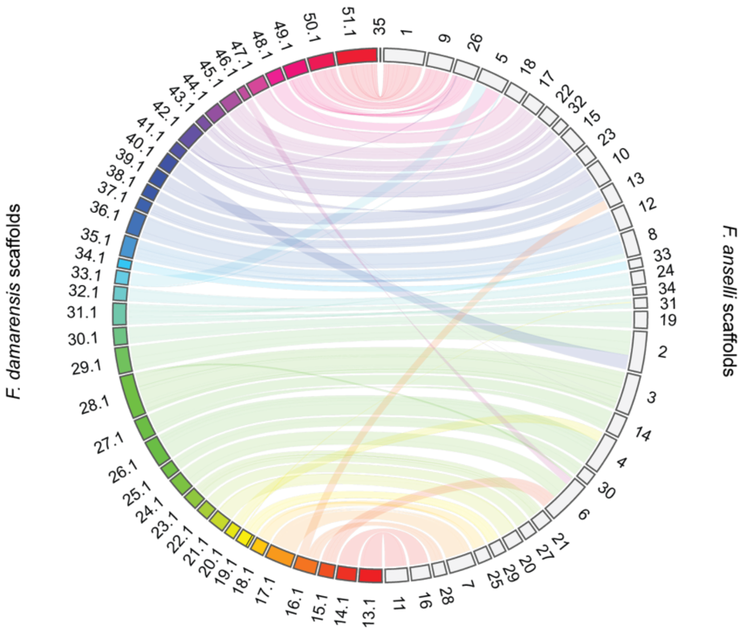

Figure S3: Circos plot depicting synteny between *Fukomys damarensis* (rainbow colored boxes) and *Fukomys anselli* (grey boxes) scaffolds for all *F. damarensis* scaffolds longer than 10Mb and for *F. anselli* scaffolds constituting 99% of the genome. The number of each scaffold is indicated, and *F. damarensis* scaffold names have been truncated to exclude the prefix "NW0229009".
