# Supplementary material for: De novo genome assembly of Ansell's mole-rat (Fukomys anselli)
Source: G3 (Bethesda). 2025 Nov 11;16(1):jkaf271. doi: 10.1093/g3journal/jkaf271 (PMC12774600; doi:10.1093/g3journal/jkaf271)
Supplement: jkaf271_Supplementary_Data [file jkaf271_supplementary_data.zip › Figure_S4_G3-2025-406291.pdf]

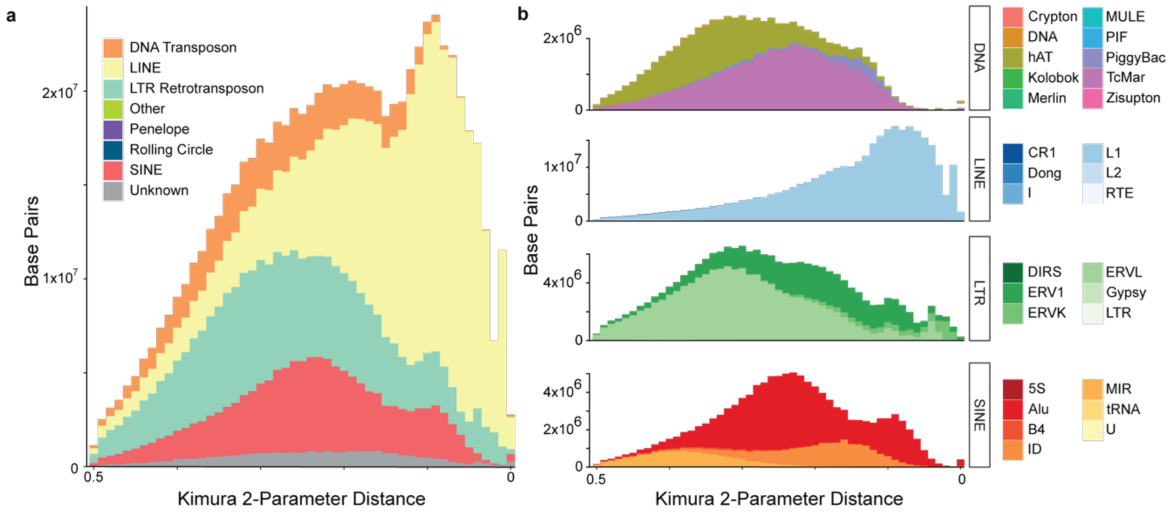

Figure S4: Kimura 2-parameter distance of transposable elements for all pooled (a) and family-resolved (b) classes in the *Fukomys anselli* genome. Some transposable element populations represented in the legends have too few occurrences to visualize at this resolution. DNA = DNA transposon; LINE = long interspersed nuclear element; SINE = short interspersed nuclear element; LTR = long terminal repeat
